# How the brain dances across critical boundaries

**DOI:** 10.1371/journal.pbio.3003950

**Published:** 2026-09-02

**Authors:** Audrey J. Sederberg

**Affiliations:** School of Physics and School of Psychological and Brain Sciences, Georgia Institute of Technology, Atlanta, Georgia, United States of America

## Abstract

The brain’s functional connectivity dynamics have been explained by a critically tuned model, but such models miss occasional reconfigurations. This Primer discusses new PLOS Biology study showing that modulatory control near a critical point can account for these rare events.

## Teaser

The brain’s functional connectivity dynamics have been explained by a critically tuned model, but such models miss occasional reconfigurations. A new *PLOS Biology* study shows that modulatory control near a critical point can account for these rare events.

A half century ago, Voss and Clarke observed that music across diverse genres exhibited a particular type of structure in the organization of pitch and amplitude fluctuations. Analysis of the energy spectrum of each of these signals showed power law dependence on frequency, with energy at a given frequency determined by the frequency raised to a power ($f^{-\beta }$) [1]. This finding was significant as such a power law indicates that there are correlations at all scales, potentially explaining why early forms of synthetic music based on simpler processes did not sound like music. Generating true power laws is not trivial: they arise when systems approach critical transitions and usually require fine-tuning. There are stochastic processes that produce $f^{-\beta }$ structure, and sounds generated from such sequences are interesting, but no listener would confuse Beethoven for fractional Brownian motion. Underneath the power law is an orchestration of patterns across scales, certainly appreciated but not mathematically understood.

Nearly a quarter century ago, power laws in the distribution of the size and the duration of activity events in neural tissue were reported, the first experimental observation of ‘avalanche’ criticality [2]. These power laws could be explained by tuning the network to a critical point, where a small event can trigger propagation through the entire system, much like a snowball can trigger the side of a mountain to fall. Since then, many additional types of criticality have been studied in the brain, such as ones in which dynamics undergo a qualitative change upon crossing a critical boundary. A new study from Pathak and Battaglia [3] suggests that simply operating at this critical boundary may not be the whole story.

**Fig 1:**
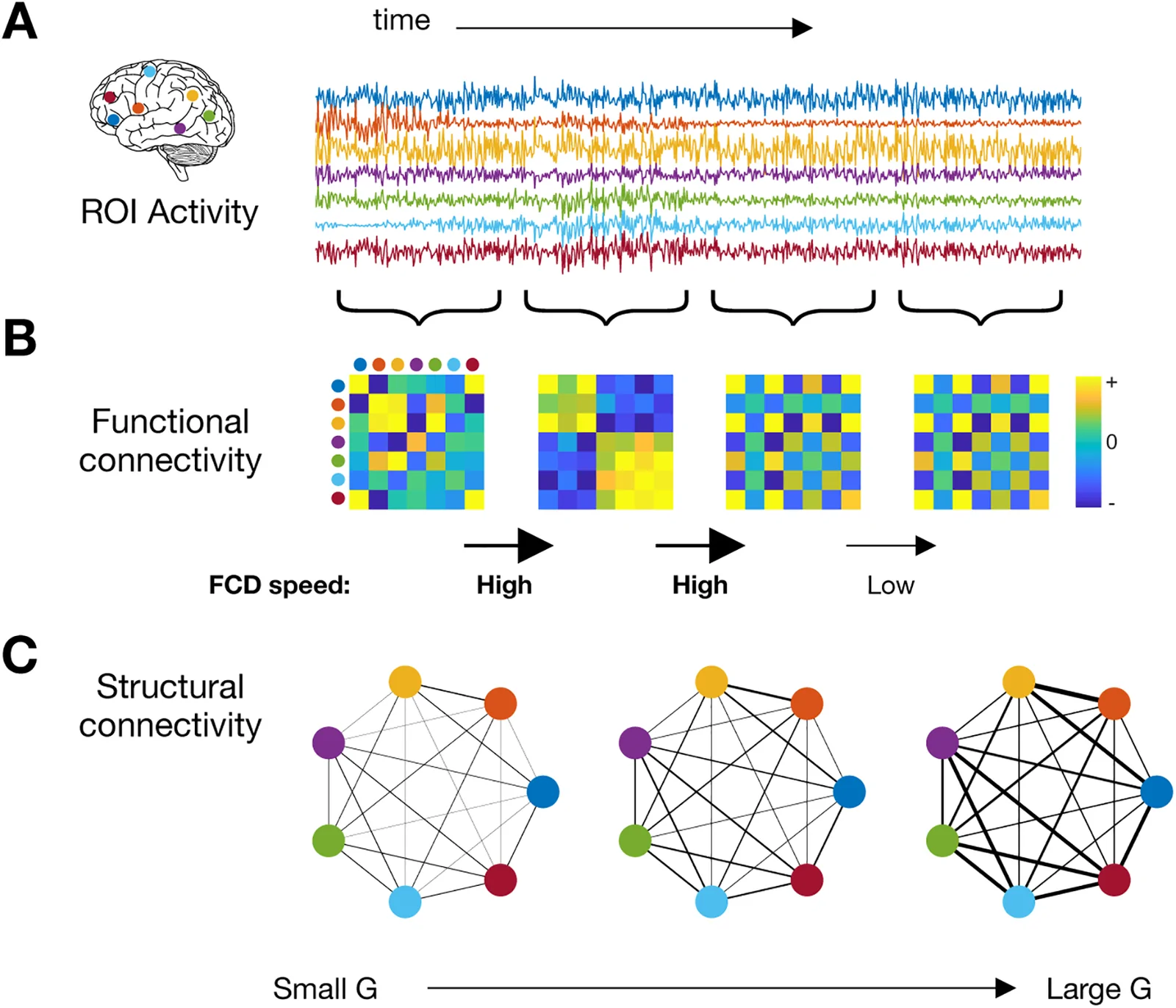
A schematic illustration of functional connectivity (FC) dynamics and modulation of global coupling strength. A: synthetic activity over time representing seven regions of interest (ROI) from a hypothetical fMRI recording. Dot locations are for illustration only. B: the FC matrix estimated within limited time windows. The structure of the FC matrix changes over time, sometimes with high speeds (first two transitions) and sometimes low (third transition). C: the anatomical connectivity of this network is fixed but can be scaled up or down. Thicker lines represent stronger interactions. At smaller G, noise can cause the network to move between activity modes, generating different FC matrices and higher speeds between successive FCs. For high G, activity will collapse into one of the available network activity patterns, and FC changes more slowly. By construction in this example, the structural connectivity supports two patterns: one, with ROI (1, 2, 3) anti-correlated with ROI (4, 5, 6, 7); the other, with ROI (1, 3, 5, 7) anti-correlated with ROIs (2, 4, 6). Neuroimaging datasets have 10s to 100s of ROIs in typical parcellations and support many more stable FCs.

In this issue, Pathak and Battaglia show how incorporating a biologically motivated modulatory dynamic can mitigate the fine-tuning problem and, along the way, better explain neuroimaging data from 100 participants from the Human Connectome Project. They begin with a curious observation: neuroimaging data reveal structure in correlations between brain areas (‘functional connectivity,’ or FC), but if examined on a minute-by-minute basis, these correlations shift between different patterns (‘FC dynamics,’ see Fig 1). Moreover, at different points in a recording, the speed at which these patterns change is not constant: there are epochs of slower and faster changes (Fig 1B). The standard models capture average FC and speeds, but they fail to explain intermittent high-speed events in the data.

Pathak and Battaglia’s innovation rests on a few key features of the standard model. Human neuroimaging data can be modeled as a collection of brain regions with anatomical connectivity taken from diffusion MRI and held fixed, but with tuneable overall coupling strength (Fig 1C). Each region receives inputs from local recurrence and synaptic drive from all other regions; these are transformed to activity by a nonlinear transfer function. Each area’s output combines with noise and feeds back through the network. These steps are controlled by a handful of constants, which parameterize the local and global interactions. Before comparing to neuroimaging data, Pathak and Battaglia explore the behavior of this model, showing that two parameters are particularly important for generating dynamic FC: global coupling and the noise strength.

There is no reason to assume that what were considered parameters are not instead variables. As a musical score is a fixed structure brought alive by a musician’s interpretation, changes in some parameters can dynamically reshape the functional modes of brain networks [4]. Pathak and Battaglia replace each of the parameters in turn with a dynamic variable and ask which (if any) of these changes improve the agreement between model and data in the distribution of FC speed. Across most participants, they find a substantial improvement, especially with dynamic global coupling. Higher order statistics of the connectivity matrices are also better explained by the modified model than the standard model. While models still operate in the vicinity of a critical boundary, they need not be at the knife’s edge. Fluctuations in the global coupling move the entire system closer or further from the boundary, which allows the system to maintain stable FC for a time, then return to an exploratory state. This prompts the authors’ hypothesis that, rather than tuning to a single critical point, the brain ‘roams’ near critical transitions, shifting between phases with higher or lower rates of change in FC.

The idea that the brain is tuned to criticality has been around for a long time but remains difficult to connect directly to practical implications for individuals. Part of this problem arises from the zoology of signatures of criticality studied across different systems, and the difficulty of proving or disproving that a system is critical, but the larger part of the problem is identifying the specific biological mechanisms that are implicated in complex statistical signatures. Pathak and Battaglia’s expanded model dances at the edge of a critical boundary, under the control of parameters that may be linked to neuromodulation. As the authors note, neuromodulatory systems change with age and in disease; their approach is a promising framework for understanding how those changes affect resting state dynamics and, ultimately, overall brain function.

Open questions remain. Despite relaxing requirements, some tuning is still required to remain near the critical boundary. Additionally, neuromodulators have complex effects on circuit function [5]. Here the global coupling strength was a single value, though the downstream effects could be quite rich given the dynamics that it gates. Accounting for details of neuromodulator projections and effects may change the model predictions.

A direct test of whether overall arousal accounts for FC speed is suggested by recent work in the mouse that demonstrated that simple measures of overall arousal account for a large fraction of resting state dynamical structure [6]. A next step for this line of work would be to monitor a participant’s arousal via pupillometry and test whether the moment-by-moment fluctuations match the predicted FC speeds.

A growing body of work is expanding our understanding of what it means for the brain to be critical, and the mechanisms in the brain that control this state. For instance, homeostatic mechanisms can return a network to a critical point [7]. Adding self-feedback can extend the repertoire of a simpler model to generate both oscillations and avalanches, explaining observations in human MEG recordings [8]. Models in which neurons are coupled to slow, low-dimensional latent variables account for multiple signatures of criticality in neural systems [9,10]. From the current study, allowing global coupling to fluctuate enables networks to shift between modes, either exploring the network space or locking into a stable configuration. Understanding how the brain controls this flexibility, and how that control changes across the life span, could have important implications for human health and wellbeing. The question is not whether the system is critical. Rather, how do we understand these statistical structures in terms of the neural mechanisms generating them? In other words, the power law is not the point: it is time to listen for the music.
